# Supplementary material for: Association between oral microbiome and seven types of cancers in East Asian population: a two-sample Mendelian randomization analysis
Source: Front Mol Biosci. 2023 Nov 21;10:1327893. doi: 10.3389/fmolb.2023.1327893 (PMC10702768; doi:10.3389/fmolb.2023.1327893)
Supplement: Supplementary file 1 [file DataSheet1.ZIP › Supplementary Materials/MR plots for tongue/tongue═╝/Ovarian cancer/pheno.3240_to_ovarian cancer_forest.pdf]

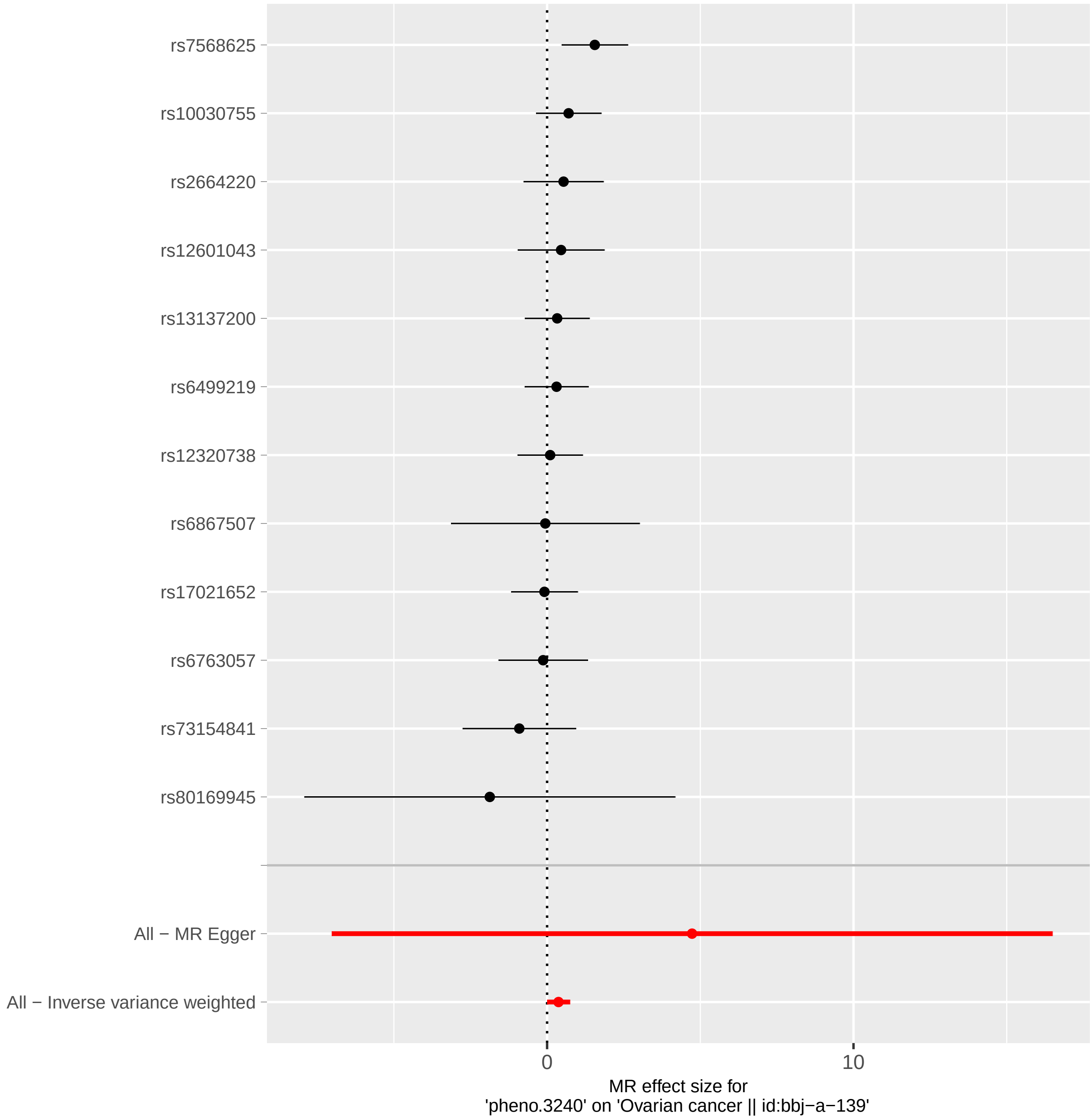

rs7568625
rs10030755
rs2664220
rs12601043
rs13137200
rs6499219
rs12320738
rs6867507
rs17021652
rs6763057
rs73154841
rs80169945
All − MR Egger
All − Inverse variance weighted
0
10
MR effect size for
'pheno.3240' on 'Ovarian cancer || id:bbj−a−139'
